# Antenatal depression: Efficacy of a pre-post therapy study and repercussions in motor development of children during the first 18 months postpartum. Study: “Pregnancy care, healthy baby”

**DOI:** 10.1016/j.jpsychires.2022.01.061

**Published:** 2022-04

**Authors:** Ricardo Tavares Pinheiro, Luciano Dias de Mattos Souza, Jéssica Puchalski Trettim, Mariana Bonati de Matos, Karen Amaral Tavares Pinheiro, Gabriela Kurz da Cunha, Bárbara Borges Rubin, Carolina Coelho Scholl, Rafaelle Stark Stigger, Janaína Vieira dos Santos Motta, Sandro Schreiber de Oliveira, Gabriele Ghisleni, Fernanda Nedel, Luciana de Avila Quevedo

**Affiliations:** a Catholic University of Pelotas, Brazil; b Federal University of Rio Grande, Brazil; c Federal University of Pelotas, Brazil

**Keywords:** Antenatal depression, Cognitive behavior therapy, Motivational interview, Motor development, Child development

## Abstract

**Aims:**

To evaluate the efficacy of brief psychotherapeutic interventions of cognitive behavioral therapy to treat antenatal depression and verify the association between interventions and motor development of infants at 3 and 18 months of age.

**Methods:**

Pre-post-intervention study nested a randomized clinical trial, both of which are extracts from a population-based cohort study of a southern Brazilian city. The major depressive episode was measured through Mini Plus, the severity of depressive symptoms by BDI-II and motor development using Bayley-III and AIMS. The follow-ups occurred during the gestational period (T2) and at 3 (T3) and 18 months (T4) after delivery.

**Results:**

Data were analyzed from 336 women in the control group (not intervened) and 108 from the group of depressed women who received intervention for antenatal depression. The effectiveness of the interventions for a major depressive episode was around 80% for both models in the two follow-up stages (3 and 18 months postpartum). In addition, the children whose mothers received intervention presented 3.7 (95% CI 0.7–6.6) points higher in Bayley-III at 3 months old when compared to the children in the control group (p = 0.01). There was no difference between the two psychotherapy models tested, both being equally effective (p > 0.05).

**Conclusions:**

We found that the brief psychotherapeutic interventions of cognitive behavioral therapy for gestational depression were effective in causing remission of the condition both in the short and long term, besides indirectly causing benefits also to the children, with regard to their motor development.

## Highlights

• CBT interventions are effective in the treatment of antenatal depression.
• CBT in pregnancy is associated with better motor development of children at 3 mths
• The addition of MI did not add to the efficacy for the antenatal depression.

## 1 Introduction

The Major Depressive Episode (MDE) is a manifestation that affects about 20% (Leung and Kaplan, 2009) of women during pregnancy, and occurs more frequently during this period when compared to other stages in life. It is often the first manifestation of a mood disorder that can have lifelong ramifications when not identified and treated. In addition, depression during pregnancy is strongly associated with postpartum depression (Silva et al., 2012).

The presence and continuation of depressive conditions during this period can have numerous consequences, such as impairments of the neurodevelopment in early childhood (Becker et al., 2016; Goodman, 2019), especially in the motor skills of babies. The motor development of children can be determined by genetic, social, and environmental factors that interact with each other in a complex way. In this context, children may either receive protection or live with risks to their development (Allen, 1993). The risk factors most related to delays in the motor domain are unfavorable socioeconomic conditions, low intellectual level of parents, prematurity, and little stimulation at home (Hamadani et al., 2010; Raniero et al., 2010; Valentini et al., 2019; Walker et al., 2011).

The literature shows that gestational and postpartum maternal depression may harm both the mother and the baby. Depressed mothers tend to be less sensitive and less available to their children's needs and they also tend to stimulate them precariously (Urizar and Muñoz, 2021; Walker et al., 2011). According to Piccinini et al. (2014), most of the time, depressed mothers are apathetic, interact little, and are not very warm with the baby, but sometimes they are intrusive. This may lead the children to not explore the environment, a factor that is very important for the development and improvement of motor skills (Piccinini et al., 2014). Thus, the remission of depression in the gestational period, which prevents this condition from extending to the postpartum period, represents an increase in the health potential of the mother-baby dyad and reinforces the importance of interventions to treat these conditions.

Although pharmacotherapy is a widely used treatment for depression at other times of the life cycle, during pregnancy its use is still controversial and often not recommended, considering the possible negative consequences for the healthy progress of pregnancy and the harm to the baby (Healy et al., 2016; Pearlstein, 2015). However, psychotherapy has been shown to be a safe treatment in this period, and the brief protocols are the most recommended due to performance, lower cost and satisfactory results. With that in mind, Cognitive Behavioral Therapy (CBT) is one of the forms of intervention that has been highlighted as effective in reducing depressive symptoms and bringing patients into remission (Okumura and Ichikura, 2014; Shortis et al., 2020; Trivedi, 2014).

It is understood that pregnancy brings with it physiological, behavioral, social and emotional changes that accentuate introspection and distancing from the external world. In this context, the inclusion of motivational interview techniques in psychotherapeutic treatments for pregnant women has been applied, despite still inconclusive effects in this stage of the life cycle and others, as well as in different contexts (Andretta et al., 2014; Holt et al., 2017). Since it is a patient-centered directive method, motivational interviewing should be able to increase the motivation for change through the exploration and resolution of ambivalences, and has been pointed out in the literature as an intervention that influences attitudes and practices of improvement in the perinatal period (Miller and Rollnick, 2002; Price et al., 2012).

In this context, maternal depression has become the focus of research and actions in maternal and child health services. Studies are increasingly recognizing the crucial role of pregnancy as a period of potential risk and an opportunity for intervention. However, few studies on maternal psychological health have been conducted in less developed countries, such as Brazil (Foley et al., 2021). Thus, the aim of this study was to evaluate the efficacy of brief psychotherapeutic interventions to treat gestational depression and measure therapeutic effectiveness at 3 and 18 months postpartum. As a secondary objective, we sought to verify the association between brief psychotherapeutic interventions by CBT during pregnancy and the motor development of infants at 3 and 18 months of age.

## 2 Methods

### 2.1 Outline

This is a pre-post-intervention study for gestational depression with a nested randomized clinical trial, both part of a population-based cohort that also followed the participants and their children at 3 and 18 months after delivery.

### 2.2 Cohort sample capture

The sampling process began in 2016 and was carried out in multiple stages, with census tracts authorized by the Brazilian Institute of Statistics (IBGE) as initial sampling units. More details of the cohort sample capture in the publication of Pinheiro et al. (2021).

**Fig. 1:**
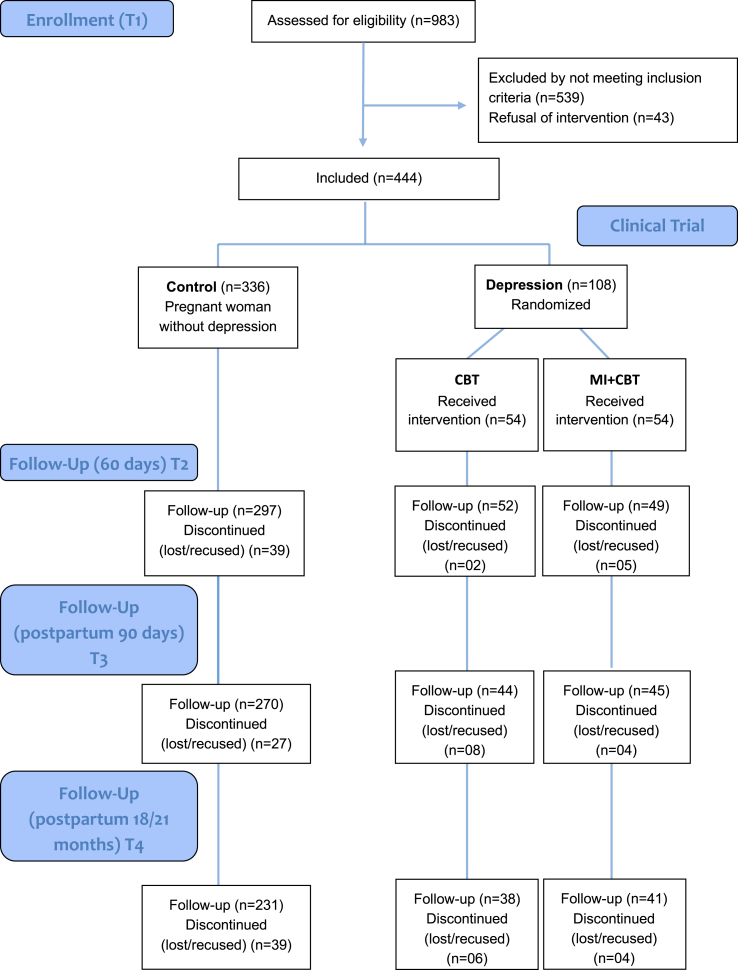
Flowchart.

The follow-up evaluations occurred at four specific stages: between the first and the second gestational trimester (T1 - pre-test) and performed at the participants' homes; the second, performed in an outpatient setting between 60 and 90 days after the first evaluation (T2), the third at 90 days after delivery (T3), and the fourth at 18 months postpartum (T4) (Fig. 1 - Flowchart).

### 2.3 Inclusion criteria for the intervention study

Pregnant women who agreed to sign the Informed Consent Form, who had a diagnosis of a current MDE (Mini Plus) and moderate to severe depressive symptoms by BDI-II and consented to participate in therapy if necessary, were included in the intervention group. For the control group, the inclusion criteria were women who were not suffering a MDE and not presenting moderate or severe depressive symptoms. In both groups, pregnant women at risk of severe suicide, who were on psychotherapeutic or pharmacological treatment and who had substance dependence (except tobacco) were excluded.

### 2.4 Inclusion criteria for randomized clinical trial (RCT - nested intervention study)

Randomization of psychotherapy models was performed for pregnant women included in the intervention group, who also agreed to participate in psychotherapeutic treatment. These pregnant women were randomly assorted into two groups that received brief psychotherapeutic intervention, both based on CBT. Thus, the first group was exposed to a six-session CBT protocol and in the other, two initial sessions of Motivational Interviewing (MI + CBT) were given in addition to the CBT protocol.

### 2.5 Psychotherapy

This study included psychotherapists (psychologists and psychiatrists) with up to five years of previous experience in mental health, without specific training in CBT, who were trained for such care. The team had training based on a manual created for the proposed intervention which aimed to educate those involved and to standardize the sessions. The therapists had weekly meetings in order to monitor and supervise those providing care.

### 2.6 Cognitive behavioral therapy protocol (CBT)

The CBT technique offered consisted of an adapted version of the manual of structured cognitive behavioral psychotherapy (Beck, 1997). This model proposed psychotherapy in six sessions that addressed distorted and/or dysfunctional thoughts (which influence the patient's mood and behavior) focusing on the pregnancy-puerperal cycle. Eligible participants received weekly sessions of 50 min of individual psychotherapy, totaling 6 sessions. Data analysis included all pregnant women who attended at least one psychotherapy session (intention-to-treat analysis).

### 2.7 Motivational interview + CBT protocol (MI + CBT)

In addition to the six-session protocol proposed for CBT, two motivational interview sessions (MI) were performed at the beginning of the psychotherapy process, configuring the MI + CBT model. The motivational interview consisted of a directive method centered around the pregnant woman that aimed to increase the intrinsic motivation for change through the exploration and resolution of ambivalences.

Five principles were explored, three specific of the CBT and two for the MI: 1) emphasis on the current interests and problems of the pregnant woman; 2) communication; 3) change occurs because of its relevance to the person's own values; 4) intrinsic motivation for change and 5) selective response to the pregnant woman's discourse in order to minimize ambivalence and motivate for change (Miller and Rollnick, 2002).

### 2.8 Measures

The MDE was evaluated using module "A" of the Mini International Neuropsychiatric Interview (Mini Plus 5.0.0 Brazilian Version) at all times (Amorim, 2000). The Mini Plus is a widely used brief diagnostic interview that evaluates the presence or absence of the main psychiatric disorders. It is divided into separated modules and each one represents a psychiatric disorder. The administration of this interview was conducted by an interviewer and all responses are dichotomous (yes/no), leading to the diagnosis of the corresponding disorder. Depressive symptoms were evaluated using the Beck Depression Inventory (BDI-II), applied in all follow-up evaluations. BDI-II was used as an indicator of severity of depressive symptoms and for classification of these moderate or very severe symptoms in the inclusion process for intervention. This is a self-reported instrument that has 21 sets of statements regarding common depressive symptoms during the previous 15 days. The responses are measured in a four-point scale (ranging from zero to three points). The interpretation of the results is based on a final score from zero to 63. The higher the score, the greater the symptom severity (Gomes-Oliveira et al., 2012).

The economic evaluation of the participants was carried out using the classification methods of the Brazilian Association of Research Companies (ABEP). This classification is based on the accumulation of material goods, the education of the householder, and conditions of the house, such as running water and paved streets. It separates participants into classes A, B, C, D or E, from the scores achieved, with the letter "A" referring to the highest socioeconomic class and "E" the lowest (Associação Brasileira de Empresas de Pesquisa, 2015). For this study, the levels were categorized as follows: A + B (highest levels), C (middle level) and D + E (lowest levels).

We assessed of the occurrence of stressful life events in the last year through the Social Readjustment Assessment Scale of Holmes and Rahe. This scale is composed of 43 stressful life events, such as divorce, childbirth, death in the family, changes in work, and others. The participants indicated if they had experienced any of these events in the last year. For each experienced event, one point was assigned (Holmes and Rahe, 1967). In this study, the events were grouped into two categories: up to 3 events and 4 events or more.

The other maternal variables such as age and schooling (in complete years and later categorized), living with a partner (yes/no), gestational trimester (1st/2nd), first pregnancy (yes/no) and use/abuse of substances such as tobacco and alcohol during pregnancy (yes/no) were collected through questions from the general structured questionnaire.

We evaluated maternal nutritional status was assessed using the Atalah. This classification was specifically designed for pregnant women, taking into account the gestational age and their current Body Mass Index (BMI) of the pregnant woman. The classification is given through the BMI curve according to their gestational age, which allows classifying the nutritional status into underweight, normal, overweight and obesity (Atalah et al., 1997).

The Bayley Scale of Infant and Toddler Development III (Bayley-III) was used to evaluate the infant motor development. This scale is administered individually and is considered a “gold standard”, estimating the development of children from 1 to 42 months of age in a safe way (Bayley, 2006). The motor subscales are some of the most used for early ages, measuring fine and gross motor skills through the observation of the children's behavior against several stimuli made by properly trained evaluators. The fine motor scale, which assesses grip skills, perceptual motor integration, motor planning, and speed, and the gross motor scale, which assesses limb and trunk posture, dynamic movements, locomotion, coordination and balance. The results are obtained regarding general motor performance through both these scales. For this study, we used the composite score of the motor development scale, in which the higher scores, as better child development.

The Alberta Infant Motor Scale (AIMS) is an observational scale for the evaluation of broad motor development. The AIMS has 58 items that inform about the children's spontaneous movement in four subscales (or postures): prone (21 items), supine (09 items), sitting (12 items), and standing (16 items). The items are presented in the form of drawings ordered according to the stages of development in each subscale and are accompanied by specific observation criteria that consider aspects of posture and weight distribution, and antigravity movements presented by the children. Each item is classified as “observed” or “not observed”, with one point being assigned for each observed item and zero for each not observed item. The raw score is obtained by the sum of points from the four subscales. The gross motor performance of the children is identified through percentiles (Valentini and Saccani, 2011). For this study, a dichotomous percentile was used as a correction for the Brazilian population, where percentile <25 percentile refers to the indication of delay in motor development and percentile ≥25 indicates normal development.

### 2.9 Outcomes

The first outcome was a MDE in the in the first 18 months postpartum, evaluated with Mini Plus, where we sought to verify whether the interventions were successful in reducing depression. Subsequently, in the RCT, we evaluated which of the two therapy models (CBT and MI + CBT) was more effective to treat depression in T3 and T4. The severity of depressive symptoms measured by the mean BDI-II was also considered as an outcome.

Another outcome analyzed was the motor development of infants at 3 and 18 months using the Bayley-III (on both occasions) and AIMS (in T3). The analysis was made comparing all who received intervention with the control group and later between the two intervention models.

### 2.10 Sample size calculation

The sample size calculation was performed using the Statcalc from the EpiInfo application, considering a power of 80% and a 95% confidence interval. For the intervention study, we considered a prevalence of 16% postpartum depression (PPD) for the control group and 32% for the exposed group (gestational depression). Thus, the highest N required was 78 pregnant women in the exposed group and 234 pregnant women in the non-exposed group (control). For the RCT, we considered a prevalence of 45% of PPD for the standard protocol (CBT) and 20% for the protocol to be evaluated (MI + CBT), with the N required of 54 pregnant women in each protocol group, according to Fleiss (Fleiss et al., 2003).

### 2.11 Data processing and analysis

The data were double-entered in EpiData 3.1 (Lauritsen JM, 2002) for checking inconsistencies, and later transferred to the Statistical Package for the Social Sciences (SPSS) (IBM, 2016) where statistical analyses were performed by simple and relative frequencies, mean and standard deviation, chi-square test (x^2^), *t*-test, ANOVA, Poisson regression and linear regression when the adjusted multivariate analysis was taken.

### 2.12 Ethical considerations

All subjects gave written informed consent for the analysis and anonymous publication of research findings. The project from which this study is linked was approved by the Research Ethics Committee of the Catholic University of Pelotas under protocol number 47807915.4.0000.5339, process number 1.729.653 and Trial registration Universal Trial Number (UTN) U1111-1227-9789.

## 3 Results

**Fig. 2:**
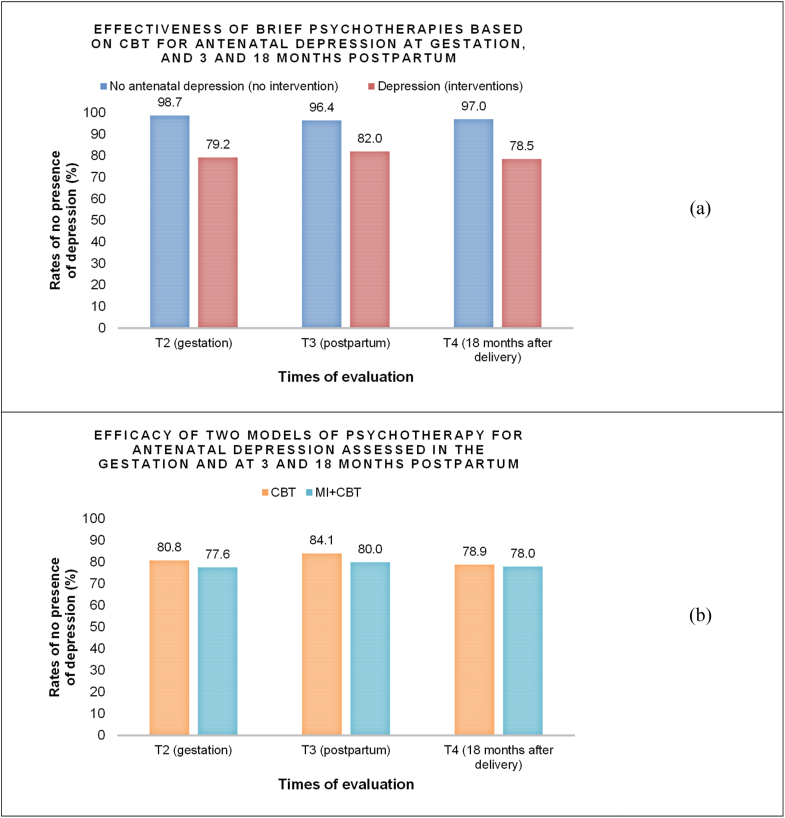
(a) Effectiveness for major depressive episode of the combined intervention models and (b) Efficacy of the randomized controlled trial for major depressive episode in the follow-ups (p > 0.05). CBT = Cognitive Behavioral Therapy; MI = Motivational Interview.

We included 444 women in the intervention study, 336 from the control group (not intervened), and 108 from the group of depressed women who received intervention for antenatal depression. Regarding the RCT nested in this intervention, the 108 depressed pregnant women were randomly allocated between the two intervention models, where half (N = 54) received psychotherapy by a CBT protocol and the other half (N = 54) received psychotherapy by a MI + CBT protocol. The effectiveness for MDE of the combined intervention models among follow-ups (T2, T3, and T4) is presented in Fig. 2(a) and the efficacy for RCT to MDE between follow-ups (T2, T3, and T4) is presented in Fig. 2(b). We observed that, in general, 80% of women had remission of depression, regardless of the time of follow-up and the protocol of intervention (CBT or MI + CBT). Such effectiveness rates are above the initial hypothesis, which were around 50%.

The characterization of the sample of the intervention study, tracing a comparison between the control group (pregnant women without depression and without intervention) and the group of pregnant women depressed during pregnancy who received intervention is presented in Table 1. The lower part of Table 1 shows the results, as a bivariate analysis, at 3 (T3) and 18 months postpartum (T4), which are: diagnosis of MDE, severity of depressive symptoms and child motor development.

**Table 1:** Characterization of the sample, control group *versus* intervention group.

VARIABLES	Control N (%)	MDE in pregnancy (CBT or MI + CBT) N (%)	p-value x^2^ or *t*-test^a^
**Age**			0.12
≤23 years	99 (29.5)	29 (26.9)	
Between 24 and 29 years	102 (30.4)	44 (40.7)	
≥30 years	135 (40.2)	35 (32.4)	
**Schooling**			0.37
≤8 years of study	64 (19.0)	24 (22.2)	
Between 9 and 11 years of study	151 (44.9)	53 (49.1)	
≥12 years of study	121 (36.0)	31 (28.7)	
**Socioeconomic level**			<0.01
A + B (highest)	116 (35.0)	17 (16.3)	
C (middle)	185 (55.9)	69 (66.3)	
D + E (lowest)	30 (9.1)	18 (17.3)	
**Live with a partner**			<0.01
No	23 (6.8)	37 (34.3)	
Yes	313 (93.2)	71 (65.7)	
**Gestational trimestrer**			0.40
1st	105 (31.3)	29 (26.9)	
2nd	231 (68.8)	79 (73.1)	
**Pregnancy planning**			<0.01
No	89 (26.5)	68 (63.0)	
Yes	247 (73.5)	40 (37.0)	
**Previous pregnancy**			0.32
No	156 (46.4)	44 (40.7)	
Yes	180 (53.6)	64 (59.3)	
**Stressful events (SRAS-Homes/Rahe)**			<0.01
≤3 events	222 (66.3)	14 (13.0)	
≥4 events	113 (33.7)	94 (87.0)	
**Depressive symptoms (BDI-II)**	6.2 (3.8)	25.7 (7.3)	<0.01
**Outcomes 3 months postpartum (T3)**			
**PPD (Mini Plus)**			<0.01
No	264 (97.8)	73 (82.0)	
Yes	06 (2.2)	16 (18.0)	
**Depressive symptoms (BDI- II)**^a^	5.9 (5.5)	16.7 (10.5)	<0.01
**Motor development (Bayley III)**^a^	103.6 (12.5)	107.0 (13.1)	0.03
**Motor development (AIMS)**			0.03
<25 percentile	51 (18.9)	08 (8.9)	
≥25 percentile	219 (81.1)	82 (91.1)	
**Outcomes 18 months postpartum (T4)**			
**MDE (Mini Plus)**			<0.01
No	224 (97.0)	62 (78.5)	
Yes	07 (3.0)	17 (21.5)	
**Depressive symptoms (BDI-II)**^a^	7.7 (7.7)	19.0 (10.6)	<0.01
**Motor development (Bayley III)**^a^	99.6 (10.4)	99.7 (10.6)	0.94
**TOTAL**	**336 (100.0)**	**108 (100.0)**	

MDE = Major Depressive Episode; CBT=Cognitive Behavior Therapy; MI + CBT=Motivational Interview + Cognitive Behavior Therapy; SRAS= Social Readjustment Assessment Scale; BDI=Beck Depression Inventory; AIMS = Alberta Infant Motor Scale.

a Mean (±sd).

Regarding depression, there was a higher presence of MDE in the intervention group in both stages of evaluation (T3 and T4), as well as in the difference in the means of depressive symptoms when compared to the control group (p < 0.05). When comparing the differences of the means between T3 and T1, the intervention group presented a higher fall in the means in relation to the control group (p < 0.01). In T3, the control group showed a reduction of 0.3 points in relation to T1, while in the intervention group the reduction was 9 points (p < 0.01). Considering the same criteria, in the evaluation at 18 months postpartum (T4) the mean of depressive symptoms from the control group increased 1.5 points in relation to T1, while there was a decrease in the group that received intervention of 7 points (p < 0.01). Thus, although the intervention group presented a mean severity of depressive symptoms greater than the control group, there was a significant reduction of these symptoms still present at T3 and T4 (Table 1).

We also observed that, when analyzing part of the sample of the intervention group that were in remission from MDE in the postpartum period, the mean number of depressive symptoms by BDI-II was 13.8 (SD ± 8.2), and the mean baseline was 25.3 (SD ± 7.9). At 18 months postpartum, the mean of depressive symptoms by BDI-II in the intervention group that presented remission of the diagnosis of depression was 16.0 (SD ± 8.6) points, still below what would be considered moderate or severe symptoms by said scale.

**Fig. 3:**
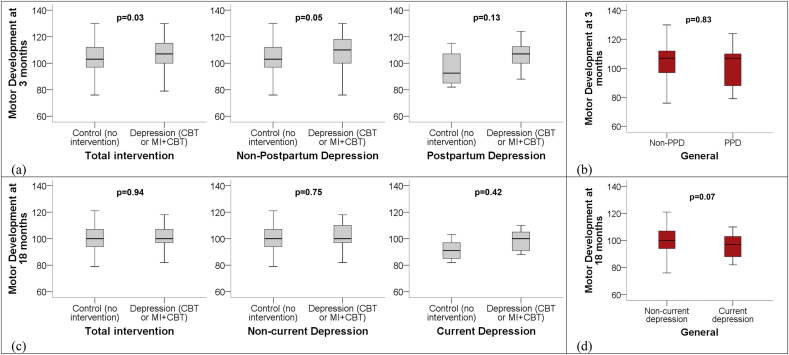
Bayley III composite scores of babies aged 3 months and 18 according to maternal gestational intervention and diagnosis of depression at times: (a) Motor performance scores distribution at 3 months according to maternal psychotherapeutic intervention during pregnancy and diagnosis of PPD; (b) Motor performance scores distribution at 3 months according to the mothers' PPD diagnosis; (c) Motor performance scores distribution at 18 months according to maternal psychotherapeutic intervention during pregnancy and diagnosis of current depression; (d) Motor performance scores distribution at 18 months according to the mothers' diagnosis of current depression. CBT = Cognitive Behavior Therapy; MI = Motivational Interview; PPD = Postpartum depression.

Fig. 3 shows the motor development of babies aged 3 months and 18 according to maternal gestational intervention and diagnosis of depression divided in: (a) motor performance scores distribution at 3 months according to maternal psychotherapeutic intervention during pregnancy and diagnosis of PPD, (b) motor performance scores distribution at 3 months according to the mothers' PPD diagnosis, (c) motor performance scores distribution at 18 months according to maternal psychotherapeutic intervention during pregnancy and diagnosis of current depression, (d) motor performance scores distribution at 18 months according to the mothers’ diagnosis of current depression. When evaluating motor development at 3 months postpartum, we verified that children whose mothers received intervention for depression during the gestational period presented better motor development when compared to children from the control group (p ≤ 0.03). This result was maintained even after linear regression analysis (Bayley-III) and Poisson regression (AIMS) adjusted for maternal age, pregnancy planning, stressful events, living with a partner, socioeconomic level, exclusive breastfeeding, first pregnancy, prematurity, low birth weight, maternal nutritional status, alcohol and tobacco use during pregnancy and diagnosis of depression at the moment. Low weight was the variable that remained associated with lower motor development; however, it did not cancel out the association between intervention and better motor development at 3 months. When translating this into numbers, the children of mothers who received the intervention presented 3.7 (95% CI 0.7–6.6) points higher in Bayley-III compared to children in the control group (p = 0.01). Regarding AIMS, the children from the intervention group were 2.7 (95% CI 1.1–6.6) less likely to present an indication of delay in the general development at 3 months when compared to children in the group without intervention (p = 0.03). In the follow-up performed at 18 months, this association was not maintained (p > 0.05).

**Fig. 4:**
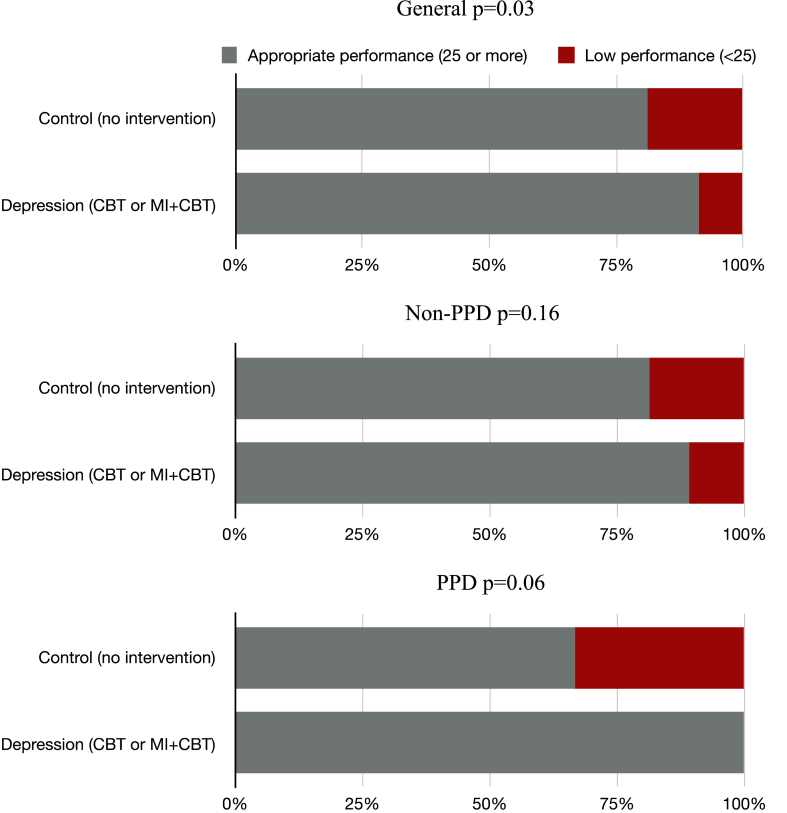
Brazilian percentile score by the Alberta Infant Motor Scale (AIMS) of infants at 3 months of age according to maternal gestational intervention and diagnosis of postpartum depression. PPD= Postpartum Depression; CBT= Cognitive Behavior Therapy; MI = Motivational Interview.

The regressions set out in the previous paragraph demanded that additional ancillary analyses were conducted. These analyses showed, at first, that postpartum depression was not associated with lower motor performance assessed by Bayley-III at that time. However, when motor development was analyzed by group, "control *versus* intervention*”*, we identified that those children whose mothers were intervened, that is, who were depressed during pregnancy and who received intervention, had better motor performance when compared to the children of mothers of the control group (p = 0.03; Figs. 3(a) and Figure 4).

In addition, a further analysis of children whose mothers did not suffer PPD, demonstrated that babies from the group of mothers who had been affected by depression but then went into remission after intervention, had better motor development when compared to the children of mothers in the control group (p = 0.05; Fig. 3(a)). Among the children of mothers with postpartum depression, we detected that those that received intervention had a tendency to have better motor skills development (p = 0.13; Fig. 3(a)).

Regarding gross motor development assessed by AIMS, we found that children whose mothers were intervened for gestational depression had lower rates of delay in the development of motor skill performance when compared to children from mothers in the control group (p = 0.03). Moreover, when analyzed according to the diagnosis of PPD, the cases with indication of delay in motor development were all in the control group, but there was no difference between the diagnosis of PPD and the gross motor development at 3 months postpartum (p > 0.05; Fig. 4).

At 18 months postpartum, we found an association between worse motor development by Bayley-III and maternal depression (p = 0.07; Fig. 3(d)). In the intervention study, there was no difference in motor performance among the children of depressed mothers who received intervention during pregnancy when compared to the children of mothers in the control group (p > 0.05; Fig. 3 (c)). Thus, the presence of depression at that stage, although it has a tendency to be associated with the worst motor development, was not explained by this child being born to a mother from the control or the intervention group.

By focusing on the RCT, we can verify that it had a satisfactory randomization of the sample, with no difference in any of the exposure variables between the CBT and MI + CBT groups (Table 2). Regarding the MDE outcome, there was no difference in the presence of the disorder at the stages of follow-up between the intervention models, as well as the severity of depressive symptoms at T3 and T4. The reduction of depressive symptoms was on average 9 points in T3 and approximately 7 points in T4, both in relation to the pre-intervention (T1), indicating a stability in the means of depressive symptomatology of women who received any of the two models of psychotherapeutic intervention (Table 2). Thus, Table 2 shows that depressed pregnant women intervened with CBT and those depressed pregnant women intervened by the MI + CBT model, not only as a result of randomization, presented similar characteristics as well as in the outcomes of a MDE, in the severity of depressive symptoms and motor development of children.

**Table 2:** Characterization of the Randomized Clinical Trial models.

VARIABLES	MDE (CBT) N (%)	MDE (MI + CBT) N (%)	p-value x^2^ or *t*-test^a^
**Age**			0.31
≤23 years	17 (31.5)	12 (22.2)	
Between 24 and 29 years	23 (42.6)	21 (38.9)	
≥30 years	14 (25.9)	21 (38.9)	
**Schooling**			0.16
≤8 years of study	16 (29.6)	08 (14.8)	
Between 9 and 11 years of study	25 (46.3)	28 (51.9)	
≥12 years of study	13 (24.1)	18 (33.3)	
**Socioeconomic level**			0.52
A + B (highest)	08 (15.7)	09 (17.0)	
C (middle)	32 (62.7)	37 (69.8)	
D + E (lowest)	11 (21.6)	07 (13.2)	
**Live with a partner**			1.00
No	19 (35.2)	18 (33.3)	
Yes	35 (64.8)	36 (66.7)	
**Gestational trimestrer**			1.00
1st	15 (27.8)	14 (25.9)	
2nd	39 (72.2)	40 (74.1)	
**Pregnancy planning**			0.84
No	21 (38.9)	19 (35.2)	
Yes	33 (61.1)	35 (64.8)	
**Previous pregnancy**			0.84
No	23 (42.6)	21 (38.9)	
Yes	31 (57.4)	33 (61.1)	
**Stressful events (SRAS-Homes/Rahe)**			0.78
≤3 events	08 (14.8)	06 (11.1)	
≥4 events	46 (85.2)	48 (88.9)	
**Depressive symptoms (BDI-II)**	25.7 (7.7)	25.8 (7.0)	0.93
**Outcomes 3 months postpartum (T3)**			
**PPD (Mini Plus)**			0.78
No	37 (50.7)	36 (49.3)	
Yes	07 (43.8)	09 (56.3)	
**Depressive symptoms (BDI- II)**^a^	16.2 (10.0)	17.1 (11.0)	0.70
**Motor development (Bayley III)**^a^	109.2 (12.5)	104.8 (13.6)	0.12
**Motor development (AIMS)**			1.00
<25 percentile	04 (50.0)	04 (48.8)	
≥25 percentile	40 (50.0)	42 (51.2)	
**Outcomes 18 months postpartum (T4)**			
**MDE (Mini Plus)**			
No	30 (78.9)	32 (78.0)	
Yes	08 (21.1)	09 (22.0)	
**Depressive symptoms (BDI-II)**^a^	19.2 (10.1)	18.8 (11.1)	0.87
**Motor development (Bayley III)**^a^	99.7 (8.4)	99.8 (12.5)	0.96
**TOTAL**	**54 (100.0)**	**54 (100.0)**	

MDE = Major Depressive Episode; CBT=Cognitive Behavior Therapy; MI + CBT=Motivational Interview + Cognitive Behavior Therapy; SRAS= Social Readjustment Assessment Scale; BDI=Beck Depression Inventory; AIMS = Alberta Infant Motor Scale.

a Mean (±sd).

## 4 Discussion

The overall effectiveness of the intervention performed with depressed pregnant women found in this study was higher than that reported by some other studies (Dixon and Dantas, 2017). The theoretical basis for CBT is described as one of the most effective for depressive symptoms throughout different stages of life. The effectiveness for brief CBT-based interventions for depression fluctuates around 50 and 60% for the perinatal period (O'Connor et al., 2016). In our study, the effectiveness, both at 3 and 18 months postpartum, was around 80%. As important as reducing the MDE is the evaluation of the continuation of therapeutic improvement. This gives us a parameter for the longevity of the benefits caused, which in the case of an intervention involving public health care, it is essential to consider the duration of the effect, since different socio demographic conditions will be present and the cost benefit for the system and for the population should be evaluated.

The analysis of the RCT indicated similarity of efficacy between the groups. The MI + CBT protocol was performed in an attempt to test other techniques in order to update and improve psychotherapeutic protocols, and it is important to highlight that the efficacy for the treatment of gestational depression and prevention of postpartum depression in the intervention groups was high. The addition of two sessions in the specific personnel training model to perform the motivational interview increases the complexity of the process. However, the effort to expand the care in two sessions did not bring the expected benefits, which makes us think that if we wish to add a greater workload and burden on the healthcare system, it will be necessary to provide more robust evidence to indicate that this increase is justified.

When an average is drawn, we cannot fail to consider that the efficacy for the diagnosis of MDE was around 80%, so 20% of our entire sample of depressed women that received intervention in pregnancy remained clinically depressed, with a median or the average corresponding to moderate up to severe symptoms. On the other hand, in the results of this study, those who went into remission presented a reduction of, on average 12 points, actually achieving a parameter similar to those that never had a diagnosis of depression (gestational and postpartum). In a language based on the cutoff points recommended by the BDI-II for the Brazilian population, they were placed in the same category as the control group, that is, without symptoms or with minimal symptoms. Thus, Mini Plus as a diagnostic criterion is strengthened, and, the BDI-II by average, provides us with a very useful measure of symptom severity in a clinical evaluation. Regarding the severity of the symptoms presented in the evolution of RCT, the same occurred, and the means fell in the same proportion both in the CBT model and in the MI + CBT model, at 3 and 18 months postpartum. Thus, one more parameter indicates that in our population adding MI was not advantageous.

When considering the high rate of women who develop depressive symptoms during the perinatal period (Acheampong et al., 2021), it is essential that interventions are effective, with results that continue throughout the first years of their children's lives. Our study also proposed to investigate as an outcome the motor development of babies at 3 and 18 months postpartum. This concern is justified by the already known impact of maternal depression on the establishment of the mother-child bond and on the neurocognitive development of these children, which has an important consequence for the dyad. Thus, such prejudice can be avoided with investment in treatments during the gestational period (Tomlinson et al., 2018).

Our results indicated that children born to mothers who were depressed during pregnancy who were also treated, presented better early motor performance when compared to children from the control group and, therefore, received no intervention. This result was not the initial expectation, since the literature leads one to believe that children born to women with gestational depression have lower motor development scores in early childhood (Gressier et al., 2020; O'Leary et al., 2019). The point is that at 3 months of age and most significantly in children whose mothers were given intervention, they had better performance, in both gross motor and fine motor skills.

Moreover, when we analyzed, separately, only those children whose mothers did not have PPD, we found that the children of women in the control group had lower motor performance than children from depressed and intervened women during pregnancy. This leads us to an important question: does having gestational depression, then being treated and going into remission in postpartum actually have a positive impact on the motor development of children? One hypothesis is that even presenting gestational depression, these women obtained during the therapeutic experience an improvement in the correction of their distorted and/or dysfunctional thoughts regarding pregnancy and motherhood and also, psychotherapy may have provided a resolution of ambivalences regarding the baby.

The findings from our study are somewhat similar to those found in the literature. Despite the lack of studies investigating the effect of psychotherapies for antenatal depression on child development, a study revealed higher child development scores (problem solving, self-regulation, and communication) of 9-month-old children of mothers with gestational depression who performed CBT when compared to children of mothers with gestational depression in usual care (Milgrom et al., 2015). Nevertheless, when evaluating them at 2 years of age, there was no difference in motor development. Despite this, children of mothers who underwent intervention showed a tendency to have higher motor development scores, favoring intervention (Milgrom et al., 2019). Related to our RCT, it is also worth mentioning that the type of model used did not interfere in the motor performance of the babies, a behavior similar to the depression outcome, both at 3 and 18 months of life of the children.

The 15-month time gap caused the group of children from mothers of the control group to reach similar levels of motor skill development to the children from the mothers in the intervention group. We question the reason why the effect of treatment clearly had repercussions on the evaluation at 3 months postpartum, but at 18 months the fact that we did not find significant differences between the control and intervention groups makes us hypothesize that other uncontrollable factors may have interfered in the process.

Finding a direct result of the greater motor development of children at 3 months of age in the intervention group gave us an expectation that this would manifest in the same way at 18 months. In any case, achieving similar levels of motor development in T4 is also positive, since gestational depression did not bring such negative consequences to the motor development of these children, reinforcing the importance of treatment during the gestational period, with good effects in the first 18 months.

According to Simcock et al. (2018), the timing of stress exposure during pregnancy may also explain child development. Higher levels of hardship during late pregnancy may harm the motor development of infants more, while this effect may not be that significant during early pregnancy. The authors revealed an improvement in the trajectory of child motor development over time, which was hypothetically explained by the children's cerebellar maturation (Simcock et al., 2018). In the same direction, Bleker et al. (2020) points to preliminary results on changes in fetal development as a consequence of maternal interventions (Bleker et al., 2020). In this perspective, a hypothesis for our findings is that the proposed intervention, which started during the second gestational trimester and concluded in the beginning of the third trimester, could have generated the improvement of these mothers at a crucial moment for the formation of the nervous system of these babies (Moore and Persaud, 2020). Simcock's proposal is related to stress, however, stress and depression have some similarities in their responses to the functioning of the central nervous system. Although still distant, this hypothesis can be better explored in future studies.

Finally, we suggest we add that maintaining a supporting team with support via telephone, internet, messaging applications and social networks, especially considering our current pandemic altered lifestyles, can in an effective and with low costs, help minimize the emotional suffering of these families in this period of the life cycle. Such facts provide a direct, constant and simplified communication between users and health service providers at all levels. Furthermore, we believe in the importance of future studies that investigate beyond developmental outcomes, with hypotheses of broader relationships of association with neurobiological outcomes of maternal intervention in children.

## 5 Limitations and strengths

As one of the limitations of the study, we highlight that the evaluation at 18 months postpartum brings a series of events that were introduced in the multivariate analysis model. However, even if some possible factors have not been controlled, we assess that the main ones involved have been conducted to analysis. Another limitation to be considered was the design of protocols with different numbers of sessions. We leave a suggestion for standardization for future replications.

Considering the strengths, we highlight the methodological rigor of the study and the carefully selected instruments. In addition, the possibility of replicating the study, based on the accessibility and detailing of the protocols and the analysis of the data exposed throughout the paper, as a suggestion of a complement to basic prenatal care.

## 6 Conclusions

This manuscript presented results related to a pre-post-intervention study and, linked to this, an RCT. On one hand, the pre-post-intervention study showed significant differences, indicating that our hypothesis of effectiveness was correct, but on the other, regarding RCT, we did not see our hypothesis corroborated. Our research contributes to the positive results that psychological monitoring during prenatal care may provide, both in the important reduction of cases of gestational depression and prevention of postpartum depression in mothers, as well as with indirect results in motor development of children. Through brief protocols, with possible sociocultural adaptation and standardization, we were able to observe a direct benefit on maternal mental health and in the motor development of children, emphasizing the importance of screening and early intervention for depression during the gestational period. In addition, the high applicability that this diagnosis and treatment model provides is reinforced, favoring reliable screening and a follow-up that is easy to monitor and can be adopted by prenatal health services.

Finally, we could say that the effort to make a therapeutic intervention for gestational depression is essential for maternal and child health, not only treating depression but also preventing PPD and future neurodevelopment deficits in children.

## Author statement contributors

R. T. Pinheiro is principal investigator and project manager. M. de Matos, K. Pinheiro, J. Motta, F. Nedel, A. Ardais G. Ghisleni, S. Oliveira and L. Quevedo authors contributed to the conception and design of the study and participated in the elaboration of the questionnaire. M. de Matos, G. da Cunha, R. Stigger, B. Rubin, C. Scholl and J. Trettim coordinated the fieldwork and participated in the acquisition of data. R. T. Pinheiro, J. Trettim, L. Souza and L. Quevedo contributed to the writing of the article and inclusion of articles in the review. All authors provided feedback on drafts of the manuscript and interpreted the results and all authors have approved the final manuscript.

## Funding and acknowledgments

This work was supported by Ministry of Health (DECIT), CNPq/Brazil (Process 401726/2015-0 APP/Call 47/2014), 10.13039/100000865Bill & Melinda Gates Foundation (INV-007186/OPP1142172) and INCT-DCEN (National Institute of Science and Technology). Also we thank all pregnant women who participated in our study.

## Declaration of competing interest

The authors declare that they have no conflict of interest.
